# The effects of an emergency department length-of-stay management system on severely ill patients’ treatment outcomes

**DOI:** 10.1186/s12873-022-00760-z

**Published:** 2022-12-13

**Authors:** Young Eun Kim, Hyang Yuol Lee

**Affiliations:** 1 Department of Emergency Medicine, Seoul St. Mary’s Hospital, 222 Banpo-daero, Seocho-gu, Seoul, 06591 South Korea; 2 College of Nursing, The Catholic University of Korea, 222 Banpo-daero, Seocho-gu, Seoul, 06591 Republic of Korea

**Keywords:** Emergency treatment, Emergency medical service communication systems, Patient care management, Length of stay, Hospitalization, Mortality

## Abstract

**Purpose:**

This study aimed to compare the length of stay (LOS) and treatment outcomes based on the application and achievement of a newly developed emergency department (ED) LOS management system for severely ill patients.

**Methods:**

Data were retrospectively collected from electronic medical records (EMRs) for the system evaluation and research purpose. The study subjects are severely ill patients whose diagnosis codes are designated by the Ministry of Health and Welfare and who visited the ED of a tertiary hospital from January to December 2019. The control group (Group 1) refers to those who have neither applied nor achieved the goal (5 hours or less) of the ED LOS management system even after it was applied, and the experimental group (Group 2) refers to those who have achieved the 5-hour goal after applying the system.

**Results:**

A total of 2034 severely ill patients applied the ED LOS management system. Group 1 included 837 patients and Group 2 included 1197 patients. Thirty days in-hospital mortality corresponded to 10.6% in Group 1 and 6.6% in Group 2 (χ2 = 10.58, *p* = .001). The total duration of hospitalization was 14.66 ± 18.26 days in Group 1 and 10.19 ± 16.00 days in Group 2 (t = 9.03, *p* < .001). Six hundred forty-two patients (76.6%) in Group 1 were discharged to their home (normal discharge) and 979 patients (81.7%) were discharged to their home in Group 2, but the discharge-as-death rate was 14.1% in Group 1 and 7.5% in Group 2 (χ2 = 29.80, *p* < .001).

**Conclusion:**

With the application and attainment of the ED LOS management system for severely ill patients, we have concluded the new system produced a lower LOS in the ED, 30 days in-hospital mortality, length of the hospitalization, mortality rate, and a higher rate of normal discharge.

**Supplementary Information:**

The online version contains supplementary material available at 10.1186/s12873-022-00760-z.

## Introduction

Emergency Department (ED) patients come to the hospital with various diseases and symptoms, and physicians must select severe patients and provide high-quality medical services at an early stage [1]. However, cultural, social, and systemic factors such as the increase in number of mild and chronic disease patients who prefer large hospitals cause emergency rooms (ERs) to overcrowd, resulting in limited ER beds and medical personnel for the severe patients who require prompt and appropriate treatment. Thus, these patients do not receive adequate medical services [2] and stay in the ER for too long.

The capacity (staff and space) of ERs can be limited; there are often several patient surges during a short time. Routinely, EDs face unexpected patient surges, so severely ill patients who need timely management and prompt care receive delayed treatment because of poor management systems of overcrowded ERs. The mortality rate in overcrowded ERs is 1.34 times higher than in non-overcrowded ERs [3]. Overcrowded ERs operate with a very limited number of medical personnel who can decide what to do for patients while discerning their symptoms; thus, late decisions lead to poor patient outcomes. Overcrowded ERs present confined spaces with staff shortages, but an excess demand of patient triage, which delays treatment of severe patients and increases patients’ time spent in the ER, which could result in increases in the mortality rate of severe patients [4, 5]. In addition, in the case of severe patients, the time spent in the ER is related to the occurrence of complications and deaths, so prompt treatment through the initial evaluation process and early transfer to the intensive care unit (ICU) is important [6]. A long stay in the ER increases a patient’s hospitalization period [7] and lowers the quality of emergency medical services, such as exposure to unexpected safety accidents, delays in diagnosis and treatment, and medical errors, leading to difficulties in emergency treatment [8–10].

A system policy that limits time was introduced in many countries to reduce the time patients spent in ERs. Australia and New Zealand introduced a system that manages patients’ ER stays and the introduction of length of stay (LOS) in the ER management system within 4 hours reduced hospital mortality [11, 12]. In the UK and Canada, systems are in place to manage the total patient time spent in the ER, from arrival at the ER to the first visit with the doctor, and from making an admission decision to discharge.

The Korean government and Ministry of Health and Welfare (MOHW) started changing the emergency management system in Korea by evaluating the mean time spent in the ER in tertiary hospitals as a quality indicator of medical service evaluation criteria. Hence, all the hospitals started managing the time spent in the ER for three major types of emergency patients (i.e., those with cerebrovascular disease, cardiovascular disease, and severe trauma) in 2009 to induce the efficient operations and prompt treatment of severe emergency patients due to concerns over the deterioration of the quality of emergency medical care resulting from lengthy ER stays. From 2019, the MOHW has selected patients with 28 diagnostic groups to evaluate the functional part of the emergency medical institution and timeliness of their service for patients with severe medical conditions, including whether they were triaged within an appropriate time and if that is reflected in the price of their medical services. According to the evaluation criteria of emergency medical institutions conducted by the MOHW in 2019, the stay time of patients with severe illness in the ER was encouraged to be less than 5 hours, which is recognized as the highest grade for the institution, and less than 6 hours for those diagnosed with severe symptoms is the minimum ER stay time to be paid for their service. However, although the MOHW evaluated all hospitals with the standard of staying in the ER as less than 6 hours for quality, prompt hospitalization of severely ill patients, the Ministry failed to provide a practical solution to reduce the time spent in the ER, leaving the responsibility to change the effective system and reduce waiting time to each hospital. In addition, the MOHW does not share outcomes with hospitals to know how the patients’ treatment performance is affected by evaluating and managing severely ill patients’ LOS, and intervention studies with hospital-based data are insufficient.

Therefore, we aimed to evaluate the effectiveness of a newly developed ER LOS management system applied in our institution and tried to provide clear empirical evidence to help manage the stay time of severe patients who visit domestic ERs in the future by comparing the treatment outcomes based on the achievement of the ER LOS management system goals while caring for patients with severe illness in our institution.

### Purpose of the study

The purpose of this study was to apply the LOS ER management system for patients with severe illness and compare the patients’ treatment performance with the residence time following the achievement of the system goal of ER discharge within 5 hours. The specific details are as follows.

1. Apply the ER LOS management system for severely ill patients and identify the characteristics of severely ill patients according to the achievement of the system’s goal.
2. Compare the application of the ER management system for severely ill patients and residence time according to the achievement of the system’s goal.
3. Compare the treatment outcomes (in-hospital death within 7 days, hospital death within 30 days, total hospital stay, type of discharge, and medical expenses) according to the application of the ER management system for severe patients and the achievement of the system’s goal.

## Methods

### Study design

This study utilized a retrospective case-control design that aimed to determine the effectiveness of applying the LOS ER management system based on electronic medical records (EMRs).

### Patient selection

This study was conducted from January to December 2019 at the Regional Emergency Medical Center of C University S Hospital in Seoul with 46 beds registered with the Central Emergency Medical Center. Among patients visiting the ER, patients with 28 diagnostic groups designated by the MOHW were selected (see Attachment 1) as seriously ill patients while satisfying four criteria (see Table 1) for evaluation of emergency medical institutions. Among the subjects selected as patients with severe symptoms from January to December 2019, the control group (Group 1) did not apply the ER stay time management system and/or did not achieve the goal, and the experimental group (Group 2) applied the system and achieved the goal.

**Table 1:** Criteria for the patient with severe illness code

Number	Criteria
1	Patient who visited ED within 48 hours from symptom onset
2	Patient who visited ED “directly” or “through outpatient”
3	Patient who admitted GW or ICU, via ED
4	Patient who diagnosed as “severe illness code,” either main diagnosis, sub diagnosis, or exclusion diagnosis

*ED* Emergency Department, *GW* General Ward, *ICU* Intensive Care Unit

Patients who had an onset time of more than 48 hours, an external route of admission (other or unknown), out-of-medical visits (chart copy, inpatients via ER), or those who died on arrival were excluded from the study.

### Research tool

#### General characteristics

As for the subjects’ general characteristics, age, sex, reason for visiting, and the means of their visit were investigated. The reasons for patients’ visits were divided into disease and trauma. The means of visitation were divided into emergency call 119 or ambulance, private car, or on foot.

#### Clinical characteristics

The level of consciousness, severity classification, and type of room entry were investigated as characteristics related to patients with severe illness. The level of consciousness was classified into alert, lethargy (response to verbal cues), confusion (response to pain), and unresponsiveness as the initial level of consciousness upon admission to the ER.

There are several methods for classifying patients’ severities, such as a method based on vital signs and consciousness and a method using a diagnosis name [13]. In this study, the severity was classified by two methods. The Korean Triage and Acuity Scale (KTAS) was used for the first severity classification. The KTAS prioritizes patients according to the severity of the patient’s urgency and type of disease, and treatment starts based on the patient’s severity. The KTAS is composed of Grade 1 (resuscitation), Grade 2 (emergency), Grade 3 (emergency), Grade 4 (semi-emergency), and Grade 5 (non-emergency). The lower the grade, the higher the patient’s severity. For the second severity classification, patients with severe disease designated by the MOHW were classified into Class 1, 2, or 3 with a medical diagnosis code (see Appendix Table A1). Class 1 includes cerebral bleeding and hemorrhage, severe trauma, massive burns, pulmonary embolism, deep vein thrombosis, adult respiratory distress with pulmonary edema, disseminated intravascular coagulation, acute renal failure, status after cardiopulmonary resuscitation, and shock. Class 2 includes myocardial infarction, cerebral infarction, mild head and abdominal injuries, gastrointestinal tract bleeding with foreign substance, bronchial bleeding with foreign substance, status epilepticus, diabetic coma, and arrhythmia. Class 3 includes poisoning, surgical disease (excluding indigitation and ileus), perinatal disease, premature baby and underweight baby, intestinitis gravis, indigitation and ileus, dismemberment, ophthalmology emergency, and urology emergency. The MOHW analyzed the mortality rate, frequency, type, and retransmission rate for each severe emergency disease group to classify patients with severe disease and divided them by reflecting the opinions of emergency medical experts. The lower the grade, the higher the risk of death and close effect of treatment in the acute phase on the patient’s prognosis.

The type of room admission refers to the type of room patients are admitted to from the ER, which is divided into a general ward or an ICU.

#### ER stay time

**Fig. 1:**
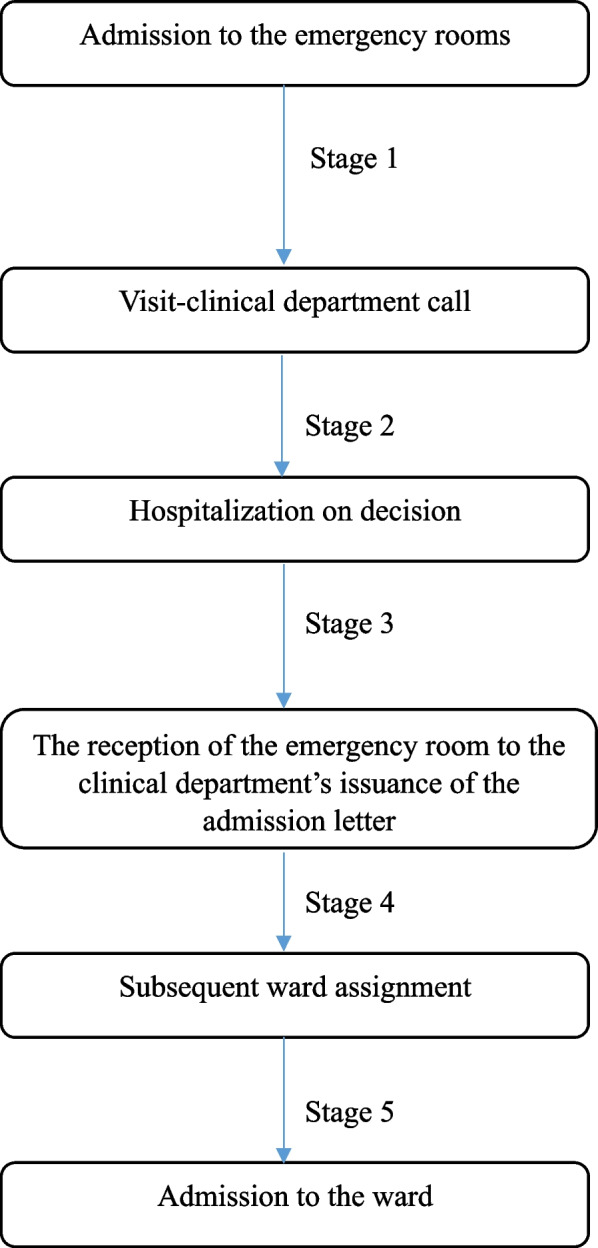
The Patient Admission Process in the Emergency Department

In this study, the time patients with severe symptoms spent in the ER was defined as the time from admission to the ER to their exit from the ER (See Fig. 1, Stage 1). In addition to the total time spent in the ER, the time required to make a decision to be admitted and waiting time for hospitalization were investigated (Stage 2). The time taken to receive a hospitalization decision refers to the time taken from the reception of the ER to the clinical department’s issuance of the admission letter (Stage 3). The waiting time for hospitalization was measured in detail by the time it took for patients to be assigned to a ward after receiving an admission letter (Stage 4) and the time it took to actually go to the ward after being assigned a ward (Stage 5). The time required was measured in minutes.

#### Patient treatment outcomes

Patient treatment outcomes consist of five items: in-hospital death within 7 days, hospital death within 30 days, total hospital stay, type of discharge, and medical expenses. Death within 7 days after hospitalization usually indicates the death from a single serious disease without other factors. Hospital death within 30 days refers to whether the patient died within 30 days of hospitalization. The total LOS was expressed in days from the date of admission to the date of discharge. The three types of discharge are classified as normal discharge (discharge to home), transfer as discharging to a nursing home or facility, and death in the hospital. Medical expenses refer to direct medical expenses that are used only for treatment, excluding hospital room fees, during the patient’s hospital stay. It was calculated as the total amount before insurance was applied.

### Research process

#### Development of a system for managing the time spent in the ER for seriously ill patients

Changing patients’ ER LOS with a new management system did not require additional costs, but building a new management system took ER staff and medical personnel’s time and effort. Nine emergency medicine specialists (medical doctors), five emergency nursing team members, and two emergency staff members participated in a meeting four times a month to develop a system for managing the time spent in the ER for severely ill patients in the emergency medical center where this study was conducted. The stay time was analyzed according to the ER inpatient treatment procedure to identify the factors of delay in staying in the ER and solve the derived problems. The institution received an average of 67,000 patients per year, of which 2300 were diagnosed with severe symptoms. Analyzing the LOS in 2018, the average stay time of patients with severe symptoms was 12.9 hours. For patients with severe symptoms, the average visit-clinical department call was 3.5 hours, the average visit-clinical and hospitalization decision was 4.3 hours, the average time to receive a hospitalization decision and subsequent ward assignment was 6.4 hours, and the length of time between receiving a ward assignment to exiting the ER was 2.1 hours on average. Based on these results, members of the meeting gathered their opinions through brainstorming and developed a new system for managing the time spent in the ER for severely ill patients in the emergency medical center. Referring to previous literature with cases of other countries, the results of evaluating emergency medical institutions by the MOHW, and the MOHW’s evaluation criteria for time management of severely ill patients, the new system goal was to leave the ER within 5 hours, and a detailed goal was set to achieve this according to the ER inpatient treatment procedure to implement the system more intensively by applying a rather complicated but detailed and specific standard:

1. The department of emergency medicine accepts patients with severe symptoms within 1 hour of visiting the ER
2. The clinical department decides on hospitalization, transfer, and discharge within 2 hours
3. Emergency staff will be assigned to a ward within 3 hours
4. Hospitalization after leaving the ER occurs within 5 hours

#### How to apply the ER LOS management system for severe patients

After developing an ER LOS management system for severe patients, the system was introduced to the emergency medical center where this study was conducted from January 2019. All staff in the hospital were notified, and all personnel working in the ER, including specialists, nurses, medical departments, and radiology departments, were educated on the purpose and method, symptoms, names of patients with severe symptoms, etc. At the hospital level, four rooms (with one bed for each room) in the general wards were secured for patients to enter.

When a patient who complains of symptoms corresponding to a severe illness visits the hospital, the nurse in the area immediately contacts an emergency medical specialist or a medical doctor (i.e., a resident of Emergency Medicine), and the emergency medical department determines whether they are a severely ill patient based on the diagnosis code set by the MOHW. If the system is activated by registering them as a severely ill patient, the patient information on the EMR patient list changes to green, and the nurse in the area delivers and shares that information with the members managing the severely ill patients. The nurse in charge of the area prioritizes the patient’s treatment over the general patients and contacts each clinician to make a quick therapeutic decision. When the clinician decides to admit the patient, the clinician can quickly assign a ward to the emergency hospital office and coordinate opinions with the nurse in charge of the assigned hospital room to leave the ER within 5 hours. During the study, one emergency medical specialist, two to three emergency medical majors, two clinical clinicians, 12 to 13 nurses, and two to three emergency staff members worked per hour. In addition, four preliminary beds for patients with severe illness in the ER were always secured and kept constant.

In this study, the ER stay time management system was applied, and the system was defined as the final goal of patients leaving the ER within 5 hours.

### Data collection methods

After receiving institutional review board (IRB) approval from Catholic University Seoul St. Mary’s Hospital, the purpose of the study was explained and agreed to by the nursing department, head of the emergency medical center, and head nurse of the emergency medical center. As this study was conducted retrospectively reviewing EMRs along with administratively collected data, the IRB committee approved the exemption of receiving patients’ consent forms. After the IRB’s approval of this study, data were retrieved retrospectively for research purposes from the hospital’s EMR system in the ED. Then, personal identifying information (i.e., name and EMR code number) was removed, and all information was anonymized for data analysis.

From January to December 2019, all patients’ data for severe medical conditions were collected through the emergency patient register, ER discharge patient register, National ED Information System’s (NEDIS) inpatient management register, and daily ER survey data.

### Data analysis methods

Statistical processing of collected data was performed using SAS (Statistical Analysis Status) version 9.4. The subjects’ general characteristics were analyzed using descriptive statistics (frequency, percentage, mean, and standard deviation), Wilcoxon rank sum test for continuous variables, and Chi-square for categorical variables.

## Results

**Fig. 2:**
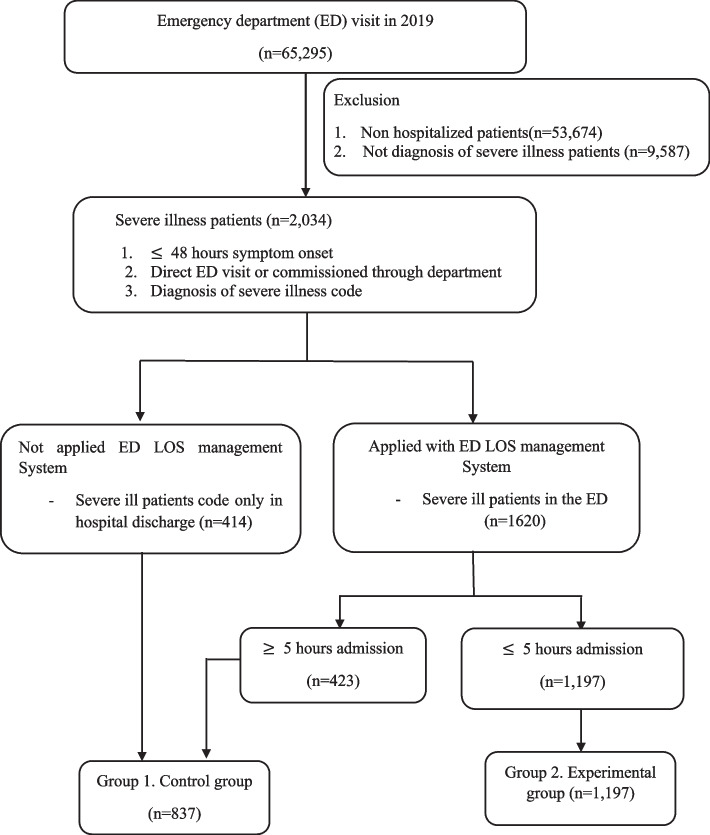
Patient Flow Diagram for Emergency Department Length of Stay Management System

During this study, a total of 65,295 patients visited the ER, 2034 of whom were severely ill; 837 patients did not apply the ER stay time management system or failed to meet their goals (Group 1) and 1197 patients applied the system and achieved their goals (Group 2) (See Fig. 2).

### General characteristics and clinical characteristics of patients with severe illness

The average age of each group was 60.17 ± 22.71 years and 61.5 ± 20.76 years, respectively, with no significant difference (t = −0.67, *p* = .501), and the ratio between men and women in the two groups was 1.3:1 and 1.6:1 (χ2 = 10.40, *p* = .001). Among the classifications of severe disease patients, the KTAS classifications for Level 3 emergency patients had 602 (71.9%) in Group 1 and 778 (65.0%) in Group 2 (See Table 2), which were the largest proportions among the three levels ([Level 1 or 2]; Level 3; [Level 4 or 5])(χ2 = 34.47, *p* < .001). For the severity classification groups divided by the MOHW’s diagnosis code, the severity was significantly different among the two groups. Group 2 includes a larger proportion of patients diagnosed with severe and emergency problems classified as Class 1 and 2 than Group 1 (χ2 = 33.47, *p* < .001). For patients’ initial consciousness, there was a significant difference among the two groups (χ2 = 7.89 *p* = .048). There were no significant differences between the two groups for patients’ profiles; patients who visited the ER for a medical emergency due to an internal issue totaled 788 (94.2%) for Group 1 versus 1119 (93.5%) for Group 2, and people who visited the ER due to a traumatic incident/accident totaled 49 (5.9%) vs. 78 (6.5%), respectively (χ2 = 0.37, *p* = .544) (see Table 2).

**Table 2:** General and clinical characteristics of participants who visited with severe illness code at ED (*N* = 2034)

Characteristics	Categories	Group 1 (*n* = 837)	Group 2 (*n* = 1197)	χ2/ t	*p*
		n (%) or M ± SD	n (%) or M ± SD		
Age (year)		60.17 ± 22.71	61.5 ± 20.76	−0.67	.501
Gender	M	449 (53.6)	728 (60.8)	10.40	.001
	F	388 (46.4)	469 (39.2)		
KTAS^a^	Level 1 or 2	103 (12.3)	268 (22.4)	34.47	<.001
	Level 3	602 (71.9)	778 (65.0)		
	Level 4 or 5	132 (15.8)	151 (12.6)		
Classification of severe illness code	Class 1	296 (35.4)	309 (25.8)	33.47	<.001
	Class 2	380 (45.4)	697 (58.2)		
	Class 3	161 (19.2)	191 (16.0)		
Initial mental state	Alert	765 (91.4)	1054 (88.1)	7.89	.048
	Response to verbal	21 (2.5)	43 (3.6)		
	Response to pain	32 (3.8)	50 (4.2)		
	Unresponsive	19 (2.3)	50 (4.2)		
Disease type	Medical	788 (94.2)	1119 (93.5)	0.37	.544
	Trauma	49 (5.9)	78 (6.5)		
Transportation	119/Ambulance	282 (33.7)	474 (39.6)	10.09	.007
	Automobile/walking	555 (66.3)	723 (60.4)		
Admission ward	GW	634 (75.8)	796 (66.5)	20.17	<.001
	ICU	203 (24.3)	401 (33.5)		

*ED* Emergency Department, *KTAS* Korean Triage and Acuity Scale, *GW* General Ward, *ICU* Intensive Care Unit

^a^KTAS Level 1: Resuscitation, Level 2: Emergency, Level 3: Urgency, Level 4: Less Urgency, Level 5: None Urgency

### Comparison of the duration of stay in the ER for patients with severe illness

The ER stay time was 965.99 ± 887.34 minutes in Group 1 and 197.73 ± 73.02 minutes in Group 2 (t = 38.43, *p* < .001). The time required to determine admission from the ER to a specialized clinical department with admission order was 386.99 ± 406.52 minutes for the control group (Group 1) and 121.98 ± 70.15 minutes for the experimental group (Group 2) (t = 26.23, *p* < .001). There was a significant difference in the time it took to secure a hospital room after patients received their hospitalization letter: 428.89 ± 771.05 minutes for Group 1 and 31.85 ± 36.59 minutes for Group 2 (t = 21.59, *p* < .001). The time it took to get to the actual room after the room was assigned was 150.86 ± 141.27 minutes for Group 1 and 47.06 ± 45.43 minutes for Group 2 (t = 19.70, *p* < .001) (see Table 3).

**Table 3:** Length of stay of participants who visited with severe illness code at ED (*N* = 2034)

Variables	Group 1 (*n* = 837)	Group 2 (*n* = 1197)	χ2/ t	*p*
	M ± SD	M ± SD		
Total length of stay in the ED (minutes)	965.99 ± 887.34	197.73 ± 73.02	38.43	<.001
Time after ED visit to admission order (minutes)	386.99 ± 406.52	121.98 ± 70.15	26.23	<.001
Time of waiting for admission (minutes)				
Time after admission order to assignment	428.89 ± 771.05	31.85 ± 36.59	21.59	<.001
Time after admission assignment to admission	150.86 ± 141.27	47.06 ± 45.43	19.70	<.001

*ED* Emergency Department; SD: Standard Deviation

### Comparison of treatment outcome of patients with severe diseases

There was no significant difference in hospital deaths within 7 days between the two groups (χ2 = 0.09 *p* = .762). However, the results of hospital deaths within 30 days showed a significant difference between the two groups (χ2 = 10.58, *p* = .001). The 30-day in-hospital mortality of Group 1 (control) was 10.6% (89/837) and that of Group 2 (experimental) was 6.6% (79/1197). In addition, the length of hospital stay totaled 14.66 ± 18.26 days in Group 1 and 10.19 ± 16.00 days in Group 2, showing significant differences (t = 9.03, *p* < .001). Among the types of discharge, normal discharge was 642 (76.6%) in Group 1 and 979 (81.7%) in Group 2, whereas deaths in the hospital were 118 (14.1%) in Group 1 and 90 (7.5%) in Group 2 (χ2 = 29.80, *p* < .001). The mean of total medical expenses were KRW 10,770,140.09 ± 19,060,781.71 for Group 1 and KRW 9,767,136.42 ± 15,809,497.95 for Group 2. Medical expenses were less in Group 2, even though the difference was not statistically significant between the two groups (t = 0.38, *p* = .699) (see Table 4).

**Table 4:** Treatment outcomes of participants who visited with severe illness code at ED (*N* = 2034)

Variables	Group 1 (*n* = 837)	Group 2 (*n* = 1197)	χ2/ t	*p*
	n (%) or M ± SD	n (%) or M ± SD		
7 days in-hospital mortality				
No	807 (96.4)	1151 (96.2)	0.09	.762
Yes	30 (3.6)	46 (3.8)		
30 days in-hospital mortality				
No	748 (89.4)	1118 (93.4)	10.58	.001
Yes	89 (10.6)	79 (6.6)		
Hospital LOS (day)	14.66 ± 18.26	10.19 ± 16.00	9.03	<.001
Type of discharge				
Discharge to home (normal discharge)	642 (76.6)	979 (81.7)	29.80	<.001
Transfer	77 (9.2)	129 (10.8)		
Death	118 (14.1)	90 (7.5)		
Total Medical Cost (won)	10,770,140.09 ± 19,060,781.71	9,767,136.42 ± 15,809,497.95	0.38	.699

*ED* Emergency Department, *LOS* Length of Stay, *M* Mean, *SD* Standard Deviation

## Discussion

This study was conducted to check the status of severely ill patients’ ER stay times and the effect of system operation to help manage future ER stays by comparing the ER stay time and patients’ treatment outcomes according to Korea’s ED LOS management system.

In the experimental group that applied the system and achieved the system’s objectives, the LOS for patients with severe medical conditions in the ER was shorter by 0.2 times compared to the control group that did not apply or did apply but did not achieve the 5-hour goal. In addition, the experimental group showed that the mean time of each clinician’s decision to hospitalize patients after visiting the ER was 4 hours shorter, the time of hospitalization after deciding to hospitalize was 6.5 hours shorter, and the duration between hospital room assignment and ER check-out was 1.7 hours shorter. In addition to the simple reduction of ER discharge within 5 hours, the detailed objectives of each stage of the ER stay management system were all achieved.

To reduce the LOS in the ER for patients with severe illness, the system goal was achieved with the participation of all department members such as nurses, emergency medicine, clinicians, and the department of administration during the initial stage of a patient’s visit to the ER. This suggests the application and achievement of the ER stay time management system was possible because the hospital’s care delivery system was improved along with the active intervention of employees.

Patients who left the ER within 5 hours by applying the ER stay time management system for seriously ill patients and achieving the system goal decreased hospital deaths, the total funding period, and hospital mortality within 30 days, and normal discharge increased. This result is consistent with prior studies that concluded longer ER stays result in increased mortality rates within 30 days [14]. In a recent domestic study, Baek and others [15] reported that the death rate of patients with shorter ER stays within 6 hours was 0.6% higher than that of patients who stayed for more than 6 hours, which indicates that prompt hospitalization of severely ill patients could be negative in terms of mortality. Han et al. [16] reported a low chance of death in patients with more than 6 hours of ER time. This is different from this study as a result of the mortality analysis rather than the overall treatment performance, which targets only the time that satisfies the LOS index for patients with severe illness and does not reflect the system’s phased time limitation.

Our study finding shows that the total length of hospital stay for severely ill patients who achieved the ER stay time system’s goal decreased by about 4.5 days compared to those who did not. This result is consistent with the previous study [7] in that ER stay time corresponds with longer total hospital LOS.

There were sicker patients in Group 2, of which patients had more KTAS scores of 1 or 2, indicating more severe illness; they also had higher rates of being admitted to the ICU, and more patients in adverse initial mental states. Total medical expenses, one of the clinical outcomes in this study, were not significantly different between the experimental group that achieved the ER stay time goal and the group that did not. Interpreting with caution, the experimental group shows 4 days shorter in hospital stay, lower 30-day in-hospital mortality, higher rates of discharge to home, and about 1,000,000 won less in mean total medical costs per patient. Hence, we can expect that limiting the triage time to less than 5 hours in the ER makes use of fewer resources and decreases medical costs, in addition to ensuring greater patient outcomes, which could be a promising result in this study.

The development and introduction of the system through a multidisciplinary approach and continuous management through regular meetings and discussions resulted in positive treatment outcomes such as shortening the time spent in the ER of severely ill patients, increasing normal discharge, and reducing in-hospital mortality. However, even if a critically ill patient leaves the ER within 5 hours by applying the ER stay time management system and achieving the goal, there are some cases where the goal cannot be achieved despite the positive effect. First, some patients with severe symptoms could not activate the ER stay time management system early because their symptoms were unknown through the patient’s initial questionnaire and physical condition. When a diagnosis of severe disease is confirmed through clinical results such as blood tests or imaging tests, it is not possible to know that the patient is seriously ill until the test results are available. However, if a diagnosis of severe disease was suspected, staff intended to proceed with priority. Second, the number of patients visiting the ER is larger than the number of medical staff. In particular, when the proportion of patients with mild symptoms is excessively large, such as on weekends and holidays, the initial treatment of patients may be delayed and the system activation of patients for severe disease can also be delayed. Therefore, it seems important not only to the hospital’s own efforts to reduce the proportion of mild patients, but also to pre-select patients from the 119 emergency service and transfer them appropriately. Third, even though the nurse in charge and department of emergency medicine quickly activated the ER stay time management system for severely ill patients, the decision to be admitted to the clinical department could be delayed. This leads to the stage where the on-call medical doctors (residents) in each department report to the on-call clinical specialist in that department after the first treatment. This phased reporting system sometimes delays the decision to hospitalize patients with severe symptoms. A concise decision-making system should be established so the emergency medicine department can directly contact the on-call specialist of each clinical department to make quicker therapeutic decisions through faster communication and consultation. Fourth, there is a problem due to the lack of hospital rooms. The emergency medical center where this study was conducted has four beds out of a total of 1368 beds to effectively apply the emergency ER management system and reduce ER stay times. For these beds, the patients can be transferred to another room within 24 hours after entering the room. However, if the reserved bed is insufficient, the existing patient’s transfer is delayed, or if the number of nurses is insufficient, it will take more than 5 hours. So, the patients may not be able to leave the ER soon enough to achieve your goal. In addition, some seriously ill patients who visit the ER require admission to the ICU, surgery, or a serious procedure. However, there is no ICU or operating room in the ER, whereas four beds are secured. Therefore, some seriously ill patients must wait in the ER because hospitalization becomes delayed due to the lack of beds in the ICU or if the operating room is not secured. Thus, practical management such as securing an ICU bed and an operating room as well as a patient bed in a general ward is urgently required to shorten the time spent in the ER for severely ill patients.

The ER stay system can be improved if we provide clear selection criteria for critically ill patients, improve ER overcrowding, establish a simple clinical and reporting system, and secure beds in ICUs or general wards for severely ill patients; then, we can expect more maximized positive effects by quickly applying the ER stay time system to more severely ill patients.

To balance the efficiency of ER management and appropriate medical treatment for patients visiting the ER, our domestic emergency medical quality evaluation added the item aiming to limit severely ill patients’ ER stay time. However, this may increase the mortality rate due to excessive efforts for ensuring shorter ER stay times [15, 16], depending on the disease, and can only induce faster hospitalization rather than hospitalization after appropriate treatment and examination. A previous study pointed out that the time goal alone is incomplete and that assessing the LOS is too simple as an indicator to judge the quality of emergency care [17]. However, the current evaluation of emergency medical institutions requires only a short stay time for severe emergency patients. The results of this study confirmed that there is a positive treatment outcome for patients when the ER stay time management system is applied, detailed goals are achieved at several stages, and the overall ER stay time is reduced. Therefore, if a specific well-designed ER system at the hospital level cannot be provided, an emergency medical evaluation based on the time spent in the ER can lead to rapid hospitalization without proper treatment and examination. Among the detailed goals of the ER stay time management system developed in this study, it would be more desirable to modify the system to reduce the time it takes for patients to be admitted to the ward after receiving a decision to be hospitalized.

The limitations of this study are as follows. First, as it relied on a short study period for patients who visited one local emergency medical center and a retrospective cross-sectional survey was conducted on a group of patients who visited one hospital, there may be a limitation in broadly interpreting the results collectively. A large-scale joint study with other medical institutions operating a system related to seriously ill patients’ LOS in the ER should be conducted. Second, diverse variables that may affect the patients’ treatment outcomes, such as the total hospital stay and cost of treatment, could not be considered overall. Patients’ demographic, social, and economic characteristics were difficult to ascertain and were not considered at all. Third, other factors that may affect ER stay time are unclear and were excluded from this study. Because many hospitals are making efforts to reduce ER overcrowding and shorten the ER stay time and overall hospital stay, these effects may affect the results, but they were not reflected in the analysis. The final goal of the ER stay time management system for severely ill patients was defined as achieving the system when they were discharged from the ER within 5 hours.

Despite these limitations, this study will have significance as the first study to determine whether the management system related to seriously ill patients’ time spent in the ER is related to the patients’ treatment outcomes such as in-hospital mortality and total hospital stay.

## Conclusion and suggestions

The ER stay time management system for severely ill patients was able to reduce severely ill patients’ time spent in the ER. If the system’s final goal of leaving the ER within 5 hours is achieved, positive study results could be obtained, with a 4.0% reduction in hospital deaths within 30 days of admission, 4.47 day reduction in total hospital stay, 6.6% reduction in in-hospital deaths, and a 5.1% increase in patients being discharged to their home (normal discharge).

This study was applied uniformly to all severely ill patients without considering patients’ conditions and clinical characteristics based on the criteria of staying in the ER for less than 5 hours, because that was the evaluation criteria of all emergency medical institutions in tertiary hospitals. The evaluation criteria may be changed for each disease group if a subsequent study related to the time spent in the ER and patients’ treatment performances is conducted according to the 28 diagnostic groups designated by the MOHW.

In addition, the ER stay time management system should introduce an effective classification system to promptly screen patients with severe disease by requiring activities of interventions through a multidisciplinary approach. Rather than only limiting the time spent in the ER, it is necessary to expand and build a larger system for efficiently managing the time spent in the ER for severely ill patients by aiming to achieve specific clinical results according to several bundles of disease groups. Furthermore, we suggest scholars further develop and manage a more comprehensive system applicable to all patients visiting the ER, not limited to patients with severe symptoms.

## Supplementary Information

**Additional file 1: Table A1**. Classification of medical diagnosis codes for severely ill patients : the Korean Ministry of Health and Welfare version.

## Data Availability

The datasets generated from patient medical records during the current study are not publicly available due to Korean medical law and personal information protection policy for our institution but are available from the corresponding author on reasonable request.
