# Supplementary material for: The effects of an emergency department length-of-stay management system on severely ill patients’ treatment outcomes
Source: BMC Emerg Med. 2022 Dec 13;22:204. doi: 10.1186/s12873-022-00760-z (PMC9745968; doi:10.1186/s12873-022-00760-z)
Supplement: Supplementary file 1 — Additional file 1: Table A1. Classification of medical diagnosis codes for severely ill patients : the Korean Ministry of Health and Welfare version. [file 12873_2022_760_MOESM1_ESM.docx]

Appendix

**Table A1**. Classification of Medical Diagnosis Codes for Severely Ill Patients : the Korean Ministry of Health and Welfare version.

| No. | Disease Group Name | | Medical Diagnosis Codes | Classification |
| --- | --- | --- | --- | --- |
| 1 | myocardial infarction | | I210∼I219 | Class 2 |
| 2 | cerebral infarction | | I6300∼I64 | Class 2 |
| 3 | cerebral parenchyma bleeding | | I610∼I629 | Class 1 |
| 4 | subarachnoid hemorrhage | | I600∼I609 | Class 1 |
| 5 | severe trauma | head injuries | S0610∼S0611, S0650∼S0651, S0660∼S0661, S0670∼S0671, S0680∼ S0681, S0200, S0201, S02180, S02181, S0620, S0621, S0690, S0691 | Class 1 |
|  |  |  | S0640∼S0641 | Class 2 |
|  |  | cervical injury | S1500~S1508 | Class 1 |
|  |  | Thoracic injuries | S2500∼S2508, S26000∼S26919, S27100∼S27219, S280, S2730~S27319 | Class 1 |
|  |  | pelvic fracture | S32820∼S32891 | Class 1 |
|  |  | Abdominal injuries | S3510∼S3558, S357, S3590∼S3598, S36100∼S36112, S3670~S3671, S36800∼S36818, S3770~S3771, S396 | Class 1 |
|  |  |  | S36400∼S36519 | Class 2 |
|  |  | injuries of lower limb | T0250~T0251, T790~T791 | Class 1 |
|  |  | - | ICISS <= 0.90 | Class 2 |
|  |  | asphyxiation | T71 | Class 2 |
|  |  | Drowning | T751 | Class 2 |
| 6 | aortic dissection | | I7101∼I7109, I7110∼I7119, I713, I715, I718 | Class 1 |
| 7 | gallbladder bile duct disease | | K8000∼K8011, K8030∼K8041, K8051, K810, K819, K830, K831 | Class 2 |
| 8 | Surgical diseases  (excluding indigitation/ileus) | | K352∼K353, K631, K650∼K659, K661 | Class 3 |
| 9 | gastrointestinal tract bleeding /foreign substance | | I8500∼I8501, I864, I983, K920∼K922, K226, K2500, K2540, K2501, K2521, K2541, K2561, K260, K262, K264, K266, T181 | Class 2 |
| 10 | Bronchial bleeding /foreign substance | | R042, R048, R049, T1740∼T1799 | Class 2 |
| 11 | Poisoning (including CO) | | T360∼T659 | Class 3 |
| 12 | Perinatal disease | | O000∼O009, O140∼O159, O4200, O4201, O4209, O4210, O4211, O4219, O4220, O4221, O4229, O4290, O4291, O4299, O450∼O459, O6000∼O6039, O800∼O809, O820∼O829, O720∼O723, O622 | Class 3 |
| 13 | premature baby / underweight baby | | P0700∼P0739, P220∼P229, P240∼P249, P360∼P369, P520∼P529, P590∼P599 | Class 3 |
| 14 | massive burns | | T3130∼T3199, T2030∼T2039, T2070∼T2079, T213, T217 | Class 1 |
| 15 | status epilepticus | | G410∼G419 | Class 2 |
| 16 | intestinitis gravis | | A830∼A870, A872, G000∼G07, A021, A227, A241, A267, A400∼A409, A410∼A414, A419, A427, B007, B377, A4188, R651, A75, A750∼A753, A759, A985, A938, B334, A77∼A79 | Class 3 |
| 17 | diabetic coma | | E1000∼E1018, E1100∼E1118, E1300∼E1318, E1400∼E1418 E160, E162, E15, E1363, E1063, E1163, E1463, E875 | Class 2 |
| 18 | pulmonary embolism/ Deep Vein Thrombosis (DVT) | | I260, I269, I802 | Class 1 |
| 19 | arrhythmia | | I441, I442, I450∼I459, I472, I480∼I489, I490, I495, I498, I499 | Class 2 |
| 20 | Adult Respiratory Distress Syndrome (ARDS)/ pulmonary edema | | J80, J81, J850∼J869, J9600∼J9699, I501, J0510, J0511 | Class 1 |
| 21 | Disseminated Intravascular Coagulation | | D65 | Class 1 |
| 22 | indigitation/ileus | | K561∼K563, K565∼K566 | Class 3 |
| 23 | dismemberment | | S480∼S489, S580∼S589, S6800∼S689, S780∼S789, S880∼ S889, S980∼S984, T050∼T059, T060∼T068, T116, T136 | Class 3 |
| 24 | Acute Renal Failure | | N170∼N179, E1128 | Class 1 |
| 25 | Ophthalmology Emergency | | H3300∼H3309, H3310∼H332, H3330∼H334, H3350∼H3358, H340∼H349, H400, H4010∼H4019, H4020∼H403, H404, H405, H406, H4080∼H409, H420, H428 | Class 3 |
| 26 | Status after cardiopulmonary resuscitation | | I460∼I469 | Class 1 |
| 27 | Urology Emergency | | N44, N4500∼N4502, N4590∼N4592 | Class 3 |
| 28 | Shock | | T794, T886, T780, T805, T782, R570, R571, R572 | Class 1 |
